# Bilateral Optical Power Meter Comparison Between NIST and CENAM*

**DOI:** 10.6028/jres.113.015

**Published:** 2008-08-01

**Authors:** I Vayshenker, X Li, D. J Livigni, J. H Lehman, J. C Bermudez, J. C Molina, Z. E Ruiz

**Affiliations:** Optoelectronics Division, National Institute of Standards and Technology, Boulder, CO 80305; Centro Nacional de Metrología, km 4,5 carretera a los Cués, Municipio El Marqués, C.P. 76241, Queretaro, México

**Keywords:** international comparison, optical fiber, optical power

## Abstract

We describe the results of a comparison of reference standards between the National Institute of Standards and Technology (NIST-USA) and Centro Nacional De Metrología (CENAM-Mexico). Open beam (free field) and optical-fiber-based measurements at wavelengths of 1302 nm and 1546 nm are reported. Both laboratories’ reference standards were compared by means of a temperature-controlled optical trap detector. Measurements showed a largest difference of less than 3.4 parts in 10^3^, which is within the combined expanded (*k* = 2) uncertainty for the laboratories’ reference standards.

## 1. Introduction

In our previous work [1–5], we reported the results of international comparisons of reference standards used in the calibration of optical power meters. Those reports describe the results that were obtained by use of open laser beams [1, 5] and optical fiber cable [2–5] at wavelengths of 1302 and 1546 nm. We also compared internal NIST laser and optical fiber power reference standards at several laser wavelengths in the visible and near infrared (NIR) [6]. In this paper, we compare the reference standards maintained by CENAM and NIST laboratories by launching optical power from an open beam and an optical fiber.

For optical fiber power meter measurements, the primary standard for each laboratory is a cryogenic radiometer described in [7, 8]. Most primary standards are designed to be used with collimated (open) beams rather than divergent beams from an optical fiber. Reference standards are typically calibrated against the primary standards by means of collimated beams, but are used with divergent beams characteristic of laser light exiting an optical fiber. A transfer standard that is insensitive to beam geometries (either collimated or divergent beam) is a very important tool for comparing reference standards.

## 2. Transfer Standard

**Fig. 1:**
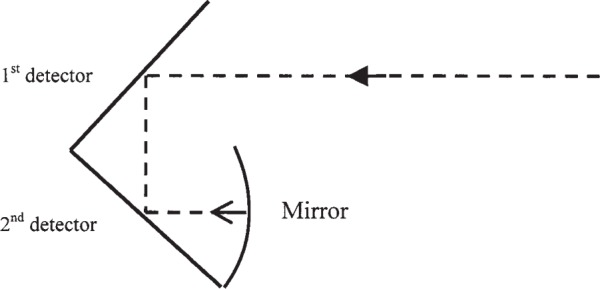
Germanium-trap detector.

For this comparison we used a transfer standard designed and built by NIST [9]. The transfer standard depicted in Fig. 1 is an optical-trap detector that consists of two germanium photodiodes and a spherical mirror. The trap detector has two Ge photodiodes 10 mm in diameter and a concave aluminum mirror of 15 mm diameter and 40 mm focal length. The mirror is coated with magnesium fluoride. The two photodiodes are oriented relative to the entrance aperture so that the principal ray of incident radiation strikes each diode once at a 45° angle of incidence and then reflects from the concave mirror back onto the photodiodes in reverse order. The photodiodes and mirror are enclosed in a thermoelectrically cooled environment. It has been shown in [10] that such a configuration provides a uniform response over the field of view and therefore requires no correction for beam geometry. A Ge-trap detector was calibrated at both national laboratories against their reference standards. The same lasers, operating at wavelengths of 1302 nm and 1546 nm, and same optical fiber cable with fiber connectors with physical contact (FC/PC) were used by both laboratories, which employed a direct substitution method for their measurements.

## 3. Measurement System

**Fig. 2:**
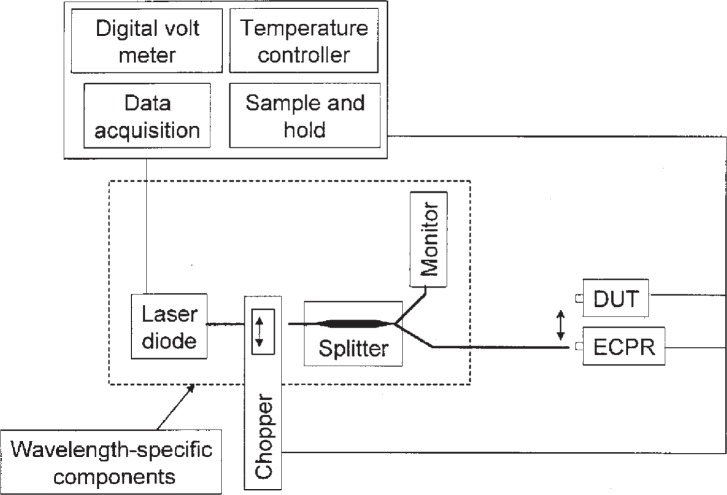
Measurement system that utilizes fiber-pigtailed laser diode sources at wavelengths of 1302 and 1546 nm.

The NIST and CENAM measurement systems are very similar; therefore in this section we describe the common measurement system used by both laboratories. The measurement system is depicted in Fig. 2; it consists of fiber-pigtailed laser diode sources at wavelengths of 1302 nm and 1546 nm, a reference optical fiber cable, and a positioning stage (see double-headed arrow) for comparing the reference and transfer (DUT) standards. The NIST measurement system is described in more detail in [11]. Both laboratories’ reference standards are electrically calibrated pyroelectric radiometers (ECPRs) that have been previously calibrated against the primary standards described in [7, 8]. The ECPR consists of a thermal detector that is covered with a gold black coating. The response of the ECPR does not depend on the wavelength of the incident radiation over the wavelength region of 1300 nm to 1550 nm [12]. The power was approximately 100 μW, or −10 dBm for both optical fiber/connector and open beam configurations.

## 4. Results of the Comparison

### 4.1 Using Optical Fiber Cable

Both participating laboratories used the same optical fiber cable with FC/PC connectors. At NIST, six measurement runs were performed with relative standard deviations of 1.2 × 10^−3^ at both wavelengths of 1302 nm and 1546 nm. At CENAM, nine measurement runs were performed with a relative standard deviation of 4 × 10^−4^ at 1302 nm and a relative standard deviation of 1 × 10^−3^ at 1546 nm. The results of the comparison are presented in Table 1.

The standard uncertainties for the optical power measurements were evaluated in accordance with ISO document standards [13]. At 1302 nm, the difference between the NIST and CENAM results was 2 parts in 10^3^, and at 1546 nm the difference was 3 parts in 10^4^ (minus sign for the difference indicates that the Ge trap detector responsivity measured by CENAM is lower than that measured by NIST). The NIST combined standard uncertainty was 2 parts in 10^3^ at 1302 nm and 2.5 parts in 10^3^ at 1546 nm, while that of CENAM’s was 1.4 parts in 10^3^ at both wavelengths of 1302 nm and 1546 nm. Table 1 provides values of relative combined standard uncertainty for both laboratories. These values were calculated by taking a square root of the sum of the squares of each laboratory’s combined uncertainty. A more detailed uncertainty analysis can be found in [11]. The observed interlaboratory differences are less than the combined standard (*k* = 1) uncertainties for the laboratories’ reference standards.

### 4. 2 Using Open Beam

Each participating laboratory used the same beam size at both wavelengths. At NIST, six measurement runs were performed with a relative standard deviation of 0.7 × 10^−3^ at a wavelength of 1302 nm and a relative standard deviation of 1.5 × 10^−3^ at a wavelength of 1546 nm. At CENAM, nine measurement runs were performed with a relative standard deviation of 6 × 10^−4^ at 1302 nm and a relative standard deviation of 5 × 10^−4^ at 1546 nm. The beam size at both wavelengths was 1.7 mm ± 0.1 mm in diameter at the 1/e^2^ intensity points. The results of the comparison are presented in Table 2.

At 1302 nm, the difference between the NIST and CENAM results was 1.4 parts in 10^3^, and at 1546 nm the difference was 3.4 parts in 10^3^ (minus sign for the difference indicates that the Ge trap detector responsivity measured by CENAM is lower than that measured by NIST). The NIST combined standard uncertainty (*k* = 1) was 1.7 parts in 10^3^ at 1302 nm and 2.3 parts in 10^3^ at 1546 nm, while that of CENAM’s was 9 parts in 10^4^ at 1302 nm and 1 part in 10^3^ at 1546 nm. Table 2 provides values of relative combined expanded uncertainties for NIST and CENAM. These values are calculated by taking a square root of the sum of the squares of each laboratory combined uncertainty. The observed interlaboratory differences are less than the relative combined expanded (*k* = 2) uncertainties for the laboratories’ reference standards.

## 5. Conclusion

This optical power meter comparison shows reasonably good agreement within the combined expanded uncertainty between NIST and CENAM. The agreement between these two laboratories is in harmony with the previous international comparisons described in [1–5]. Such comparisons are important to establish a worldwide consistency in measurements of optical power for optical telecommunications.

## Figures and Tables

**Table 1:** Results of NIST and CENAM comparison using the optical fiber cable

Source wavelength (nm)	difference (%)	combined expanded (*k* = 2) uncertainty (%)
1302	0.20	0.48
1546	−0.03	0.56

**Table 2:** Results of NIST and CENAM comparison using using open beam

Source wavelength (nm)	difference (%)	combined expanded (*k* = 2) uncertainty (%)
1302	−0.14	0.38
1546	−0.34	0.50
